# Trends in antipsychotic use among children and adolescents in Germany: a study using 2011–2020 nationwide outpatient claims data

**DOI:** 10.3389/fpsyt.2023.1264047

**Published:** 2023-12-12

**Authors:** Michael Dörks, Christian J. Bachmann, Maike Below, Falk Hoffmann, Lena M. Paschke, Oliver Scholle

**Affiliations:** ^1^Department of Health Services Research, Carl von Ossietzky University Oldenburg, Oldenburg, Germany; ^2^Department of Child & Adolescent Psychiatry, Ulm University, Ulm, Germany; ^3^Department of Child and Adolescent Psychiatry, Children’s Hospital Wilhelmstift, Hamburg, Germany; ^4^Department of Prescription Data, Central Research Institute of Ambulatory Health Care, Berlin, Germany; ^5^Department of Clinical Epidemiology, Leibniz Institute for Prevention Research and Epidemiology – BIPS, Bremen, Germany

**Keywords:** adolescents, antipsychotics, children, pediatrics, psychiatry, pharmacoepidemiology

## Abstract

**Introduction:**

We aimed to provide an update on trends in antipsychotic (AP) use among children and adolescents in Germany.

**Materials and methods:**

Based on nationwide outpatient claims data from Germany, we conducted a cross-sectional study. For each year from 2011 to 2020, we determined the prevalence of AP use, defined as the proportion of children and adolescents with at least one AP dispensation. We evaluated trends in AP use by age, sex, and AP class (typical vs. atypical). Additionally, we assessed trends in the specialty of AP prescribers and the frequency of psychiatric diagnoses among AP users.

**Results:**

Overall, data from more than 12 million children and adolescents were included for each calendar year (2011: 12,488,827; 2020: 13,330,836). From 2011 to 2020, the overall prevalence of pediatric AP use increased from 3.16 to 3.65 per 1,000, due to an increase in use of both typical APs (from 1.16 to 1.35 per 1,000) and atypical APs (from 2.35 to 2.75 per 1,000). The largest increase in AP use was found among 15- to 19-year-old females, with an increase from 3.88 per 1,000 in 2011 to 7.86 per 1,000 in 2020 (+103%), mainly due to rising quetiapine use (from 1.17 to 3.46 per 1,000). Regarding prescribers’ specialty, the proportion of APs prescribed by child and adolescent psychiatrists increased during the studied period (2011: 24.8%; 2020: 36.4%), whereas prescriptions by pediatricians (2011: 26.0%; 2020: 19.9%) and general practitioners (2011: 18.0%; 2020: 12.4%) decreased. Risperidone was the most commonly used AP in males, and quetiapine was the leading AP in females, each with the highest prevalence in 15- to 19-year-olds. In male risperidone users in this age group, the most frequent diagnosis was attention-deficit/hyperactivity disorder (50.4%), while in female quetiapine users it was depression (82.0%).

**Discussion:**

Use of APs among children and adolescents in Germany has continued to increase over the last decade. The sharp increase in AP use among 15- to 19-year-old females, which is largely due to an increased use of quetiapine, is remarkable. Potential reasons for this increase—e.g., limited access to psychosocial treatments—should be carefully analyzed. Also, the introduction of more restrictive prescribing guidelines might be considered.

## Introduction

Previous research indicates a rise in diagnosis and treatment of psychiatric disorders among children and adolescents in recent decades (1). Further studies suggest an increase in mental health problems, particularly among female adolescents and in terms of internalizing problems such as anxiety and depression (2, 3). It has been shown that this increase coincides with an increased use of psychotropic drugs (4, 5).

Antipsychotics (APs) were originally developed for the treatment of severe psychiatric disorders like schizophrenia and bipolar disorder but are now increasingly used for the treatment of restlessness, agitation, anxiety, sleep disorders, and other off-label indications (6). Particularly in children and adolescents, the off-label use of APs is common (7, 8). Even though an off-label use is often unavoidable in pediatric care, it potentially increases the risk of adverse events (9–11). While knowledge about the safety and effectiveness of AP use in pediatric patients is limited (12, 13), some studies suggest that children are at a higher risk of some adverse drug effects compared to adults, e.g., regarding extrapyramidal symptoms and metabolic abnormalities (14). Further, AP use among children and adolescents might be associated with type 2 diabetes mellitus, seizures, cardiovascular events, and unexpected death (15–18).

Despite these potential risks, studies from various countries have observed a questionable increase of AP use among children and adolescents in the last decades (19–25). This gives cause for concern and has already led to deprescribing initiatives in some countries in order to promote best practice in AP prescribing and to reduce the use of AP in children and adolescents (26–28).

In the absence of recent data for Germany, we aimed to evaluate trends in AP use among publicly insured children and adolescents in Germany, based on a 10 years observation period from 2011 to 2020.

## Materials and methods

We conducted an observational study with cross-sectional analyses in the calendar years 2011–2020 using routinely collected healthcare data from Germany.

### Data source and study sample

We used nationwide outpatient claims data from all inhabitants with statutory health insurance (SHI) for secondary data analysis. The data contain all outpatient prescriptions and diagnoses of individuals with SHI who visited an SHI-authorized physician at least once per year. SHI-insurees account for about 87%, i.e., approximately 72 million people of the total German population (29). Claims and prescription data were analyzed on behalf of all Associations of Statutory Health Insurance Physicians by the Central Research Institute of Ambulatory Health Care in accordance with §295 Social Code Book V (Sozialgesetzbuch V, SGB V).

In Germany, a physician of any specialty is permitted to prescribe APs, even if guidelines recommend that pharmacotherapy should be initiated by a child and adolescent psychiatrist. Furthermore, there are no monitoring programs or peer-review models like in the US, and AP prescription is exclusively limited to physicians (unlike in the US, where professionals other than physicians are able to prescribe APs). Prescriptions are coded according to the German modification of the World Health Organization’s Anatomical Therapeutic Chemical (ATC) classification system. Diagnoses are coded according to the German modification of the International Statistical Classification of Diseases and Related Health Problems, 10th revision (ICD-10 GM). For each calendar year from 2011 to 2020, we included data from children and adolescents aged ≤19 years with valid information on sex.

### Use of APs

AP use included outpatient prescriptions of APs dispensed in a pharmacy with an ATC code in the pharmacological subgroup “antipsychotics” (N05A), except lithium (N05AN01). For each calendar year from 2011 to 2020, we considered all AP dispensations with a prescription date between January 1 and December 31, respectively. We classified the APs used during the study period into typical (benperidol, bromperidol, chlorpromazine, chlorprothixene, droperidol, flupentixol, fluphenazine, fluspirilene, haloperidol, levomepromazine, melperone, perazine, perphenazine, pimozide, pipamperone, prothipendyl, thioridazine, tiapride, zuclopenthixol) and atypical (amisulpride, aripiprazole, asenapine, cariprazine, clozapine, loxapine, olanzapine, paliperidone, quetiapine, risperidone, sertindole, sulpiride, ziprasidone, zotepine) APs according to Kalverdijk et al. (30).

### Psychiatric diagnoses and specialty of the prescribing physician

Psychiatric diagnoses were selected according to clinical relevance. They were identified based on ICD-10 GM codes recorded in the same calendar year as the AP dispensation. The specialty of the prescribing physician was derived from the physician’s lifelong identification number on the prescription.

### Data analyses

For each calendar year from 2011 to 2020, we determined the prevalence of AP use by calculating the proportion of children and adolescents with at least one outpatient AP dispensation (per 1,000). As denominator, we used the number of individuals with at least one physician contact in the respective calendar year, which allowed full flexibility in defining the age groups. To check the robustness of our results, we conducted a sensitivity analysis, using the number of all individuals insured by SHI (according to the KM6 statistics of the Federal Ministry of Health) as the denominator (31).

Trends in the use of APs were determined overall and stratified by sex, AP class (typical vs. atypical), and by age group (<5, 5–9, 10–14, 15–19 years) separately in males and females. The age groups with the highest prevalence of AP use in females and in males in 2019 and 2020 were additionally analyzed with respect to the following: we first evaluated trends in the use of the four most commonly dispensed APs in the selected age and sex groups (on the 5th level of the ATC). Among users of the most commonly dispensed AP substance in each of the aforementioned age and sex groups in 2019 (i.e., the last pre-pandemic year), we then determined the frequency of outpatient psychiatric diagnoses in the same year. Where appropriate—i.e., for APs typically prescribed in divided dosage forms (e.g., tablets)—we additionally determined what strength (i.e., content of active ingredient per dosage form) of this AP was prescribed to these users.

## Results

Overall, the data comprised information from more than 12 million children and adolescents in each calendar year (2011: 12,488,827, 2020: 13,330,836).

### AP use overall and by age and sex

**Figure 1:**
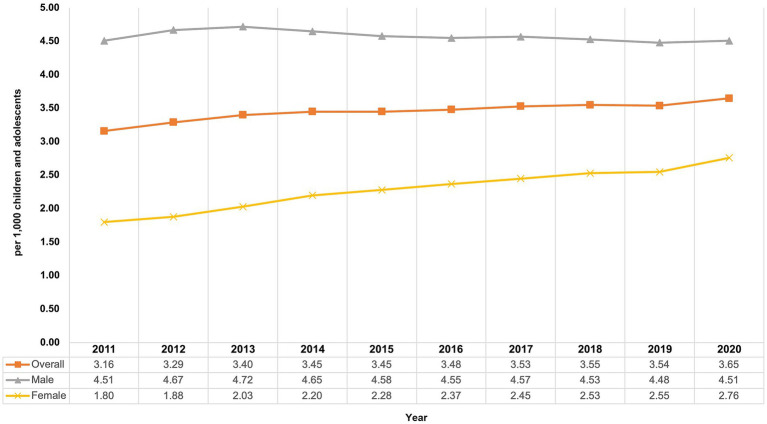
Prevalence of antipsychotic use among children and adolescents from 2011 to 2020 in Germany, overall and by sex.

From 2011 to 2020, the overall prevalence of AP use among children and adolescents increased from 3.16 to 3.65 per 1,000 children and adolescents (relative increase: +15.5%; Figure 1).

From 2011 to 2020, the prevalence of typical AP use increased from 1.16 per 1,000 to 1.35 per 1,000 and the prevalence of atypical AP use increased from 2.35 per 1,000 to 2.75 per 1,000 children and adolescents. During the study period, the prevalence of atypical AP use was about twice as high as that of typical AP use.

**Figure 2:**
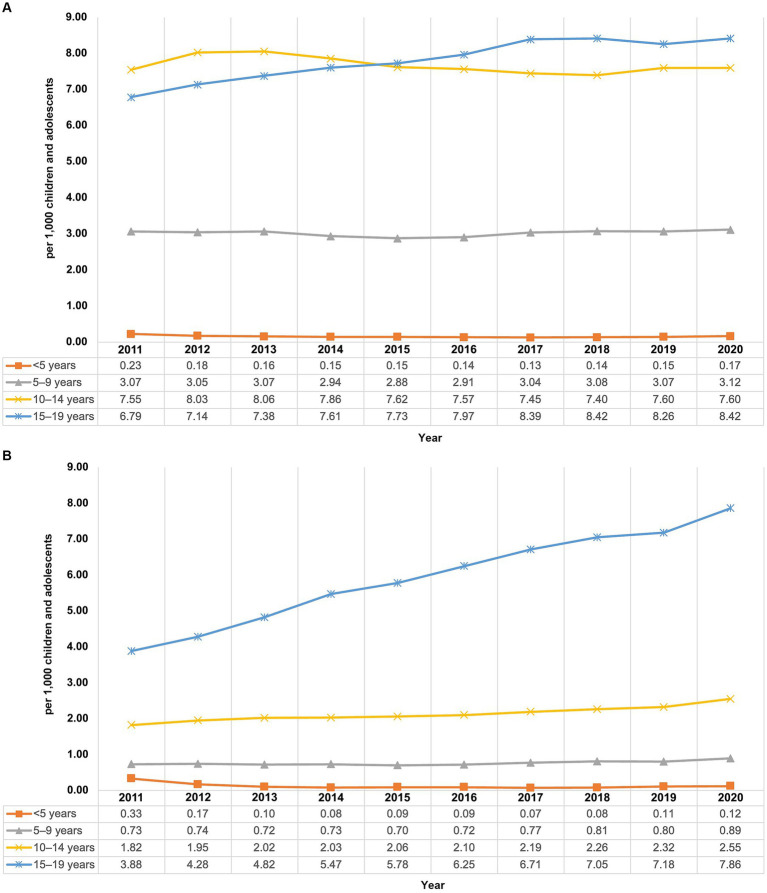
Prevalence of antipsychotic use in males **(A)** and females **(B)** in children and adolescents from 2011 to 2020 in Germany, by age group.

Stratification by age and sex showed differences between the sexes and varying trends in the prevalence of AP use depending on the observed group. Prevalence of AP use in boys remained quite stable with 4.51 per 1,000 children and adolescents at the beginning as well as at the end of the study period, whereas the prevalence in girls increased (Figure 1) from 1.80 per 1,000 in 2011 to 2.76 per 1,000 in 2020 (+53.3%). From 2011 to 2014, the highest prevalence of AP use was found in 10- to 14-year-old boys, and from 2015 to 2020 it was highest in 15- to 19-year-old boys (Figure 2). The lowest prevalence was found in the youngest age group (<5 years), with nearly identical values for girls and boys with no noticeable change throughout the study period.

In children aged 5–9 years, males (2020: 3.12 per 1,000) had an about 3.5-fold higher prevalence than females (2020: 0.89 per 1,000), and it remained stable over the whole study period. Among males aged 10–14 years, the prevalence of AP use fluctuated slightly between 2011 and 2020 with no noticeable increasing or decreasing trend over the study period (range: 7.40–8.06 per 1,000). Among females of the same age group, the prevalence increased over the study period (+40%; from 1.82 to 2.55 per 1,000). In the age group 15–19 years, increases for the prevalence could be observed from 2011 to 2020 among males (+24%; from 6.79 to 8.42 per 1,000) and females of the same age (+103%; from 3.88 per 1,000 to 7.86 per 1,000). Among males aged 15–19 years, the prevalence of AP use increased until 2017 and remained stable thereafter, whereas in females of the same age group, it steadily increased during the study period without reaching a plateau.

### Further information about selected age groups in males and females

**Figure 3:**
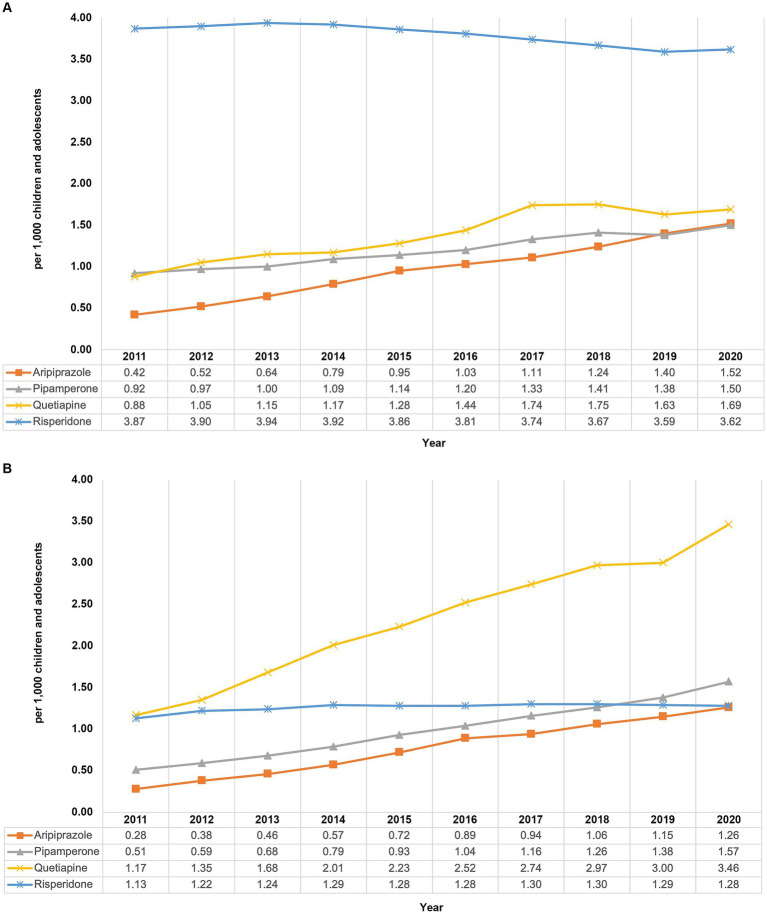
Prevalence of use of risperidone, quetiapine, pipamperone, and aripiprazole from 2011 to 2020 among 15–19-year-old males **(A)** and females **(B)**.

At the end of the study period, males and females in the age group 15–19 years had the highest prevalence of AP use and they showed increasing trends between 2011 and 2020. We therefore obtained further information about these two subgroups. Throughout the study period, risperidone was the most commonly used AP in males aged 15–19 years (Figure 3A) and quetiapine was the most commonly used AP in females aged 15–19 years (Figure 3B). In contrast to risperidone use in boys of this age group, which decreased slightly from 3.87 per 1,000 in 2011 to 3.62 per 1,000 in 2020, the use of quetiapine in females of this age group showed a steep rise from 1.17 per 1,000 in 2011 to 3.46 per 1,000 in 2020 (+196%), with the sharpest increase from 2019 to 2020. The use of quetiapine in boys also increased, but to a lesser extent and only until 2017, and the use of risperidone in girls was roughly stable. In both sexes, the use of pipamperone and aripiprazole showed permanent gradual increases until 2020 without reaching a plateau.

In male recipients of risperidone aged 15–19 years (in 2019), the most frequently coded outpatient diagnosis was attention-deficit/hyperactivity disorder (50.4%), followed by conduct disorder (29.9%) and autism spectrum disorder (27.8%; Table 1). In female quetiapine users of the same age (in 2019), the most frequent outpatient diagnoses were depression (82.0%), personality disorders (40.8%), and anxiety disorders/emotional disorders (39.3%).

**Table 1:** Outpatient psychiatric diagnoses among adolescent (age 15–19 years) male users of risperidone and female users of quetiapine in 2019.

Diagnosis	Male users of risperidone (*n* = 5,987)	Female users of quetiapine (*n* = 4,961)
Organic, including symptomatic disorders (F00–F09)	185 (3.1%)	70 (1.4%)
Eating disorders (F50)	69 (1.2%)	679 (13.7%)
Sleeping disorders (F51, G47)	103 (1.7%)	232 (4.7%)
Personality disorders (F60–F69)	1,015 (17.0%)	2,024 (40.8%)
Mental retardation^a^ (F70–F79, F84.4)	1,526 (25.5%)	151 (3.0%)
Autism spectrum disorder (F84.0/1/5/8/9)	1,663 (27.8%)	126 (2.5%)
ADHD (F90, F98.8)	3,018 (50.4%)	681 (13.7%)
Conduct disorders (F90.1, F91, F92)	1,788 (29.9%)	652 (13.1%)
Tic disorders (F95)	322 (5.4%)	35 (0.7%)
Mental and behavioral disorders due to psychoactive substance use (F10–F19)	493 (8.2%)	783 (15.8%)
Psychotic disorders (F20–F22, F25)	469 (7.8%)	392 (7.9%)
Bipolar disorders (F30, F31)	79 (1.3%)	267 (5.4%)
Depression (F32, F33, F41.2, F43.2)	1,285 (21.5%)	4,070 (82.0%)
Other mood disorders (F34, F38, F39)	117 (2.0%)	354 (7.1%)
Anxiety disorders/emotional disorders (F40, F41, F93)	864 (14.4%)	1,951 (39.3%)
Obsessive-compulsive disorder (F42)	252 (4.2%)	301 (6.1%)
Post-traumatic stress disorder (F43.0/1/8/9)	443 (7.4%)	1,459 (29.4%)
Any of the above-mentioned diagnoses	5,639 (94.2%)	4,853 (97.8%)

ADHD, attention-deficit/hyperactivity disorder; ICD-10, International Statistical Classification of Diseases and Related Health Problems, 10th revision. Diagnoses included those in the outpatient sector coded as “assured” in 2019. The same person can have more than one diagnosis.

a The authors are aware that the term “mental retardation” is outdated. Nevertheless, the term is used in this table as it is the official ICD-10 GM nomenclature for diagnoses from chapter F7.

Of all quetiapine-containing packages dispensed to 15- to 19-year-old females in 2019 (*n* = 20,043), 30% had a tablet strength of 25 mg, 34% of 50 mg, 11% of 100 mg, 8% of 150 mg, and 16% of 200 mg or higher.

### Overall AP prescribing by physicians’ specialty

**Figure 4:**
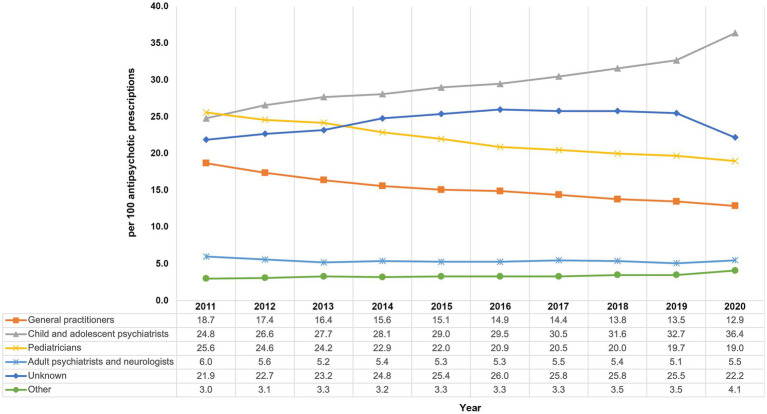
Trends in the prescribing of antipsychotics by physicians’ specialty from 2011 to 2020 among children and adolescents in Germany.

Except for 2011, where pediatricians were the most common prescribers, most APs were prescribed by child and adolescent psychiatrists with 24.8% in 2011 and 36.4% in 2020 (Figure 4) with a steadily increasing share. The proportions of prescriptions by pediatricians (2011: 25.6%, 2020: 19.0%) and general practitioners (2011: 18.7%, 2020: 12.9%) decreased throughout the study period. AP prescribing by adult psychiatrists and neurologists remained rather stable on a low level (2011: 6.0%, 2020: 5.5%). Equally, prescribing by physicians with “other” or “unknown” specialty remained stable over time. As—due to technical reasons—the “unknown” specialty label is usually used for child and adolescent psychiatric/ adult psychiatric outpatient units based at hospitals, the proportion of prescriptions issued by psychiatrists is probably even higher than stated above.

## Discussion

From 2011 to 2020, the overall prevalence of AP use among children and adolescents in Germany has continued to increase. The most striking trend regarding AP use was in females aged 15–19 years, where the prevalence of AP use doubled over the study period—mainly due to an increased use of quetiapine. In 15–19-year-old males and females (i.e., the groups with the highest use) treated with the most commonly dispensed AP (risperidone and quetiapine, respectively), the most frequently recorded diagnoses were attention-deficit/hyperactivity disorder and depressive disorders, respectively.

### Overall trends in AP use

Although knowledge about the safety of AP use in pediatric patients is still limited (12, 13), an increase of AP use in children and adolescents has been reported (19–25). For Germany, there have been only data on trends in pediatric AP use up to 2012 (25). The authors analyzed AP use in young patients ≤19 years based on claims data from one German SHI. In that study, the frequency of AP use increased from 0.23% in 2005 to 0.32% in 2012. Our study showed that the rising overall trend in pediatric AP use has continued until 2020.

Even though the reported frequency of AP use varies between countries (e.g., due to different methodologies and health system differences), similar trends of increasing AP use in children and adolescents have been observed in other Western countries (30, 32–37). A study from the UK based on the Clinical Practice Research Datalink (CPRD) database included all children and adolescents aged 3–18 years registered in the database between 2000 and 2019 (33). The authors reported that the annual period prevalence of AP use rose from 0.06% in 2000 to 0.11% in 2019. Thus, the prevalence in the UK study was at all times lower than the prevalence we found; however, this might be due to the fact that the CPRD database only contains prescriptions by general practitioners. In a Dutch study (37), the authors analyzed 84,828 AP prescriptions of children and adolescents aged 0–19 years between 2005 and 2015, derived from a large community pharmacy-based prescription database. The overall prevalence of AP use ranged from 0.72% in 2005 to 0.90% in 2015 and is thus more than twice as high as in our study.

While the prevalence of prescribed/dispensed medication should be interpreted with care and cannot be considered as overprescribing in general (24), the similarity of these trends is remarkable and the potential drivers of these trends should be carefully examined, and—if necessary—addressed. Actually, there are precedents for efforts to establish rational use of APs in children and adolescents and, where appropriate, reduce use for non-psychotic disorders (26, 27, 38). In the US, monitoring programs and peer-review models have already shown to improve the quality and reduce the prevalence of pediatric AP use (27, 38–40). Trend studies from the US found that, along with declining pediatric use of APs since 2008, there was an increase in the proportion of users with evidence-supported indications, psychosocial interventions, and metabolic monitoring for side effects (36, 41, 42).

There have been reports of an increase in atypical AP use along with a decrease of typical AP use for children and adolescents as well as adults (30, 43, 44). From 2011 to 2020, the period we considered, we found a slight increase in both, typical and atypical AP use. This is of concern as typical APs might primarily cause anticholinergic and extrapyramidal adverse events. These adverse drug effects might also appear with atypical APs, although to a lesser extent. Atypical APs may notably cause marked weight gain and hyperlipidemia, possibly resulting in metabolic syndrome (6, 45).

### AP use by age and sex

Boys are known to be more frequently treated with APs than girls. The authors of a Dutch study found a higher prevalence of AP use in boys over all study years and in all age groups (30). This is in line with our results, however, we found that the overall increase in AP use was primarily attributable to an increased use in 15–19-year-old girls (until 2020) and boys (until 2017; stable thereafter). Among females in this age group, the prevalence of AP use doubled during the study period and—in contrast to males in this age group—did not reach a plateau, but continuously rose. Particularly with the beginning of the COVID-19 pandemic and its implications in 2020, the prevalence of AP use increased more sharply from 2019 to 2020 than in previous years among females aged 10–14 and 15–19 years. It is known that today’s youth are more prone to mental health problems than previous generations (3), and it is quite conceivable that an event like the COVID-19 pandemic and the associated containment measures might have contributed to this increase in AP use. Yet, the study period we considered is too short to assume any association and, in this respect, the years after 2020 would be essential to draw any conclusion.

The increasing trends in AP use among female adolescents aged 15–19 years were mostly driven by use of quetiapine. Mental health problems have continuously increased among young people in recent decades, especially among female adolescents and regarding internalizing problems (2, 3). Depression and anxiety disorders/emotional disorders—typical diagnoses for internalizing symptoms—were among the most frequently coded psychiatric diagnoses among female adolescent users of quetiapine in our study. This suggests that quetiapine has been used to treat internalizing symptoms.

Whether the increase in quetiapine use in Germany was due to a rising burden of mental disorders, to a compensation of lacking psychotherapy capacities, or to other reasons, has to be considered in further research. At least from 2009 to 2018, the prevalence of outpatient guideline-based psychological therapies for children and adolescents has not changed markedly in Germany (46). Other factors that may contribute to the sharp increase in quetiapine use include encouraging off-label prescribing by pharmaceutical companies (47, 48) and the introduction of generic products to the German market in 2012. The impact of the introduction of generic quetiapine in Germany is difficult to assess without further investigation. As prescription drugs are available at no cost for children until 18 years of age in Germany, and as there are no direct prescription restrictions regarding quetiapine by German SHI providers, the cost of quetiapine is not expected to substantially influence physicians’ prescribing decisions. A previous study indicates that the prevalence of quetiapine use among 0–19-year-olds in Germany already increased substantially from 2005 to 2014 (44), which may be a sign of a rather low influence of generic quetiapine. The potential impact of the introduction of generic quetiapine should be evaluated in future research by including more data years prior to 2012.

Future research should also investigate whether the APs which were increasingly used over time in our study (quetiapine, aripiprazole, and pipamperone) were prescribed as an adjunct treatment (i.e., augmentation) of antidepressant therapy in treatment-resistant depression. The evidence on the benefits of this strategy in adolescents is limited and systematic reviews warn about possible adverse effects (49, 50).

Risperidone was often used by males aged 15–19 years with outpatient diagnoses of ADHD or conduct disorders in our study. This is in line with other studies showing that APs in children and adolescents are mainly prescribed to treat aggressive, impulsive, and hyperkinetic behavior associated with ADHD, autism, and intellectual disability (9, 21, 25, 37, 43, 51). Risperidone is approved for short-term treatment (up to 6 weeks) of persistent aggression in patients older than 4 years with sub-average IQ. This might explain the higher prevalence of any AP use in boys, as physical aggressive behavior is more prevalent among boys than among girls (37, 51). Short-term risperidone use may reduce aggression and conduct problems in children and youths with disruptive behavior disorders, however, there is also evidence that this intervention is associated with considerable weight gain (52).

The most frequently recorded outpatient diagnoses for 15–19-year-old females treated with quetiapine in our study were depression, personality disorders and anxiety/emotional disorders. Also in adults, an increasing use of quetiapine has been reported, together with a high proportion of off-label use (53, 54). Almost two thirds of quetiapine-containing packages dispensed to female adolescents in our study had a tablet strength of 50 mg or lower. Assuming that patients should take one tablet per day, this is considered low-dose quetiapine use (≤50 mg/day), which further indicates off-label prescribing. Recently, it has been shown that risks of metabolic worsening and major adverse cardiovascular events (in adults) are increased even with low-dose quetiapine use (55, 56). Due to the sharp increase in use and the lack of data for this vulnerable group, the safety of quetiapine use in children and adolescents should be further evaluated. In addition, the introduction of monitoring programs—such as those implementing more restrictive prescribing guidelines or education for prescribers—might be considered.

### Specialty of the prescribing physicians

We found that along with the overall increase in AP use, specifically the proportion of prescriptions by child and adolescent psychiatrists increased, while prescribing by pediatricians and general practitioners decreased. This finding might reflect a tendency in the German healthcare system to shift the initiation of a new treatment regime with an AP to child and adolescent psychiatrists.

### Strengths and limitations

A major strength of this study is the large sample size representing almost 90% of the general population and all persons covered by SHI in Germany. In contrast to primary data studies, there was no risk for potential non-responder or recall bias. Further, some limitations of our study have to be mentioned. First, due to the nature of the underlying data, we were only able to consider outpatient drug dispensations and diagnoses of SHI-insurees with at least one physician contact in the respective calendar year and the data did not include information about hospitalized or privately insured patients. As mentioned earlier, our data source covers all persons insured with a SHI, i.e., almost 90% of the German population. It has been shown that privately insured children and adolescents in Germany have a higher socioeconomic status, however, there were no substantial differences in the frequency of use of drugs from the “nervous system” group compared with those covered by SHI (57). Therefore, we assume that our results are representative regarding the prevalence of use of outpatient APs among all children and adolescents in Germany. Secondly, it should be noted that routine data generally do not allow a direct link between diagnosis and prescription. Thirdly, we do not know how long the dispensed APs were taken by the patients, or if they were taken at all. Fourthly, as we used the number of individuals with at least one physician contact in the denominator, we might overestimate the prevalence. However, our sensitivity analysis (using the total number of individuals covered by SHI) revealed the same striking trends in the older age groups as in the main analysis.

## Conclusion

Our results show that AP use among children and adolescents in Germany has continued to increase over the last decade. First and foremost, the sharp increase in AP use among females 15–19 years of age, which is largely due to an increased use of quetiapine, is remarkable. This raises concerns, since the benefit–risk ratio of off-label quetiapine use in children and adolescents is uncertain. Therefore, the reasons for the increase—e.g., limited access to psychosocial treatments or the introduction of generic quetiapine to the German market—should be critically examined and, if appropriate, the introduction of prescribing guidelines of a more restrictive nature could be considered.

## Data availability statement

The data analyzed in this study is subject to the following licenses/restrictions: the data were analyzed on behalf of the regional associations of Statutory Health Insurance Physicians in Germany (ASHIP). Due to the sensitive and confidential nature of data on outpatient drug prescriptions and claims, the authors are not permitted to publish the data. Access to the data is restricted to researchers and requires the consent of the ASHIPs. Requests to access these datasets should be directed to datenanfrage@zi.de.

## Ethics statement

Ethical review and approval was not required as our study used routinely collected anonymized data.

## Author contributions

MD: Conceptualization, Data curation, Methodology, Writing – original draft. CB: Conceptualization, Formal analysis, Methodology, Writing – review & editing, Data curation. MB: Conceptualization, Formal analysis, Methodology, Writing – review & editing, Data curation. FH: Conceptualization, Methodology, Formal analysis, Writing – review & editing. LP: Conceptualization, Data curation, Formal analysis, Methodology, Writing – review & editing. OS: Conceptualization, Formal analysis, Methodology, Writing – review & editing, Project administration.
